# Acute Coronary Syndrome and Rheumatic Disease

**DOI:** 10.3390/jcm14051490

**Published:** 2025-02-23

**Authors:** Andrew P. Hill, Shaikh B. Iqbal, Brian C. Case, Ananth A. Shankar, Ilan Merdler

**Affiliations:** 1 Section of Cardiology, MedStar Washington Hospital Center and Georgetown University, Washington, DC 20007, USA; andrew.p.hill@medstar.net; 2 Section of Internal Medicine, MedStar Union Memorial Hospital and Georgetown University, Washington, DC 20007, USA; shaikh.b.iqbal@medstar.net; 3 Section of Interventional Cardiology, MedStar Washington Hospital Center, Washington, DC 20010, USA; brian.c.case@medstar.net; 4 Essen Health Care, Bronx, NY 10461, USA; ashankar@essenmed.com

**Keywords:** acute coronary syndrome, autoimmune, inflammation, ischemia, rheumatic disease

## Abstract

Patients with rheumatic disease and immune disorders have been noted to show an earlier development of atherosclerosis and to present with acute coronary syndrome. These diseases disproportionately affect women, and patients frequently have a higher number of comorbidities and other risk factors. Inflammation has long been known to play a role in the development of coronary artery disease. Early studies have shown some benefit in treatment targeting inflammation. While this has not been realized for all populations, there remains potential in treatment with targeted and individualized therapies. Especially since these diseases are associated with a worse prognosis, management benefits from the multidisciplinary expertise of cardiologists, rheumatologists, and other providers. However, the prevention and treatment of underlying rheumatic disease remains essential. This review will seek to highlight prior studies and future directions in the treatment of acute coronary syndrome in patients with rheumatologic disease.

## 1. Introduction

**Figure 1:**
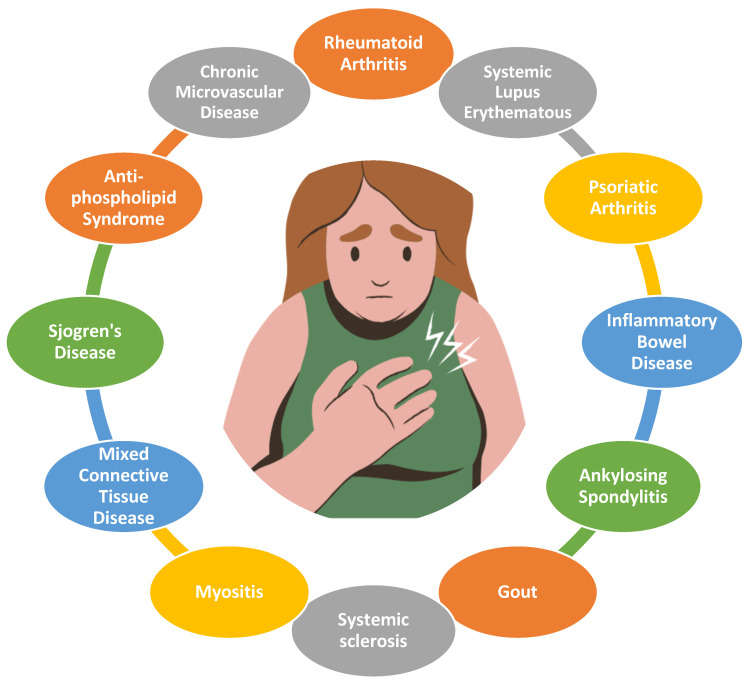
Central illustration. Acute coronary syndrome and rheumatic diseases.

Cardiovascular disease continues to be a leading cause of death [1]. Patients with rheumatic and immune disorders have been shown to have a high rate of subclinical atherosclerosis, as well as an increased presentation of acute cardiovascular events, as shown in Figure 1 [2,3]. Additionally, over the past several years, autoimmune and rheumatic disorders have continued to increase in prevalence [4]. There are a number of different factors that lead to this elevated risk—including the higher prevalence of comorbidities associated with cardiovascular disease, medications used to treat the primary rheumatic disorder, and underlying inflammation. The residual cardiac risk attributed to rheumatologic diseases is above and beyond that conferred by traditional comorbid cardiac risk factors [5]. In addition, inflammation has been shown to play a role in the development and manifestation of cardiovascular disease [6]. This includes acute coronary syndrome and has been associated with a worse prognosis in a variety of different disease states [7]. American guidelines have suggested that treatment with disease-modifying anti-rheumatoid drugs, biologic medications, and other immune modulating therapies can be considered to decrease the risk of cardiovascular events [8]. Further risk evaluation is recommended in the European guidelines with similar preventative treatments as the general population [9].

While there is a higher prevalence of atherosclerotic cardiovascular disease and other conditions such as hypertension and heart failure, less has been published about the association between rheumatic disease and acute coronary syndrome. The management of these patients is complex and benefits from the multidisciplinary expertise of cardiologists, rheumatologists, and other providers. Our review aims to focus on this specific link between inflammatory disease and the development of acute coronary syndrome in addition to current and future treatment strategies.

## 2. Methods

In this narrative review, we sought to provide an insight into the relationship between rheumatic disease and immune disorders and acute coronary syndrome, as well as to highlight the current literature and future directions for the prevention and treatment of acute coronary syndrome in patients with rheumatic disease. To identify and retrieve relevant articles, a comprehensive search of the PubMed database was conducted using a combination of Medical Subject Heading (MeSH) terms and search words related to rheumatic diseases and acute coronary syndrome. The MeSH and search terms included “acute coronary syndrome”, “ACS”, “STEMI”, “myocardial infarction”, “NSTEMI”, “gout”, “chronic inflammatory conditions”, “rheumatoid arthritis”, “rheumatic disease”, “psoriatic arthritis”, “psoriasis”, “systemic lupus erythematous”, “lupus”, “SLE”, “inflammatory bowel disease”, “IBD”, “Crohn”, “ulcerative colitis”, “ankylosing spondylitis”, “ankylosing spondyloarthritis”, “axial spondyloarthritis”, “systemic sclerosis”, “myositis”, “dermatomyositis”, “mixed connective tissue disease”, “MCTD”, “Sjogren”, “antiphospholipid syndrome”, “APLS”, and “chronic microvascular disease”. The initial search was limited to English-language articles published within the past 5 years, with exceptions for rheumatoid arthritis, psoriatic arthritis, systemic lupus erythematous, and inflammatory bowel disease due to their robust literature. 

## 3. Pathophysiology

The pathophysiology of atherosclerosis in rheumatic diseases is complex and multifactorial, as seen in Table 1. Chronic inflammation, a hallmark of rheumatic conditions, plays a central role in the development and progression of atherosclerosis. Persistent inflammation leads to endothelial dysfunction, increased oxidative stress, and dysregulation of the immune system, all of which contribute to the formation and destabilization of atherosclerotic plaques. This inflammation is associated with the persistent activation of immune cells, including T-cells and macrophages, which results in the release of pro-inflammatory cytokines (e.g., Tumor necrosis factor alpha (TNF-α), Interleukin 6 (IL-6), and Interleukin-1 beta (IL-1β)) and can lead to deleterious effects through various signaling pathways.

Briefly, TNF-α can lead to cell apoptosis in myocytes and endothelial cells [10,11]. It also recruits neutrophils and macrophages in addition to upregulating nitric oxide production and calcium dysregulation. IL-6 is produced by macrophages, monocytes, endothelial cells, vascular smooth muscle cells, and fibroblasts, and upon activation, myeloid cells can also produce IL-6 with other pro-inflammatory cytokines, creating a significant positive feedback loop [12,13]. IL-6 can lead to T-cell apoptosis inhibition, inflammatory cell recruitment, and the inhibition of regulatory T-cell differentiation, and is associated with higher levels of C-reactive protein (CRP) [14,15]. The IL-1 family is important in the innate immune response, and it consists of the main pro-inflammatory cytokines implicated in cardiovascular disease. IL-1β is the main circulating type of IL-1. IL-1β, the main circulating type, can be produced by monocytes, macrophages, and neutrophils, and it causes inflammation through IL-6 signaling as well as by increasing acute phase reactants, such as CRP [16] and ([17], p. 1).

The mechanism of acute coronary syndrome can be associated with inflammation. As forementioned, pro-inflammatory cytokines are involved throughout the development of atherosclerosis, and both local and systemic inflammation are important in this process. Plaque rupture without systemic inflammation can occur, but frequently, activated macrophages, T-cells, and pro-inflammatory cytokines are involved in the accumulation of a central lipid core and the development of a thin fibrous cap [18]. This collagen-poor weakened fibrous cap is prone to rupture in the setting of disturbed flow and abnormal shear stress.

The vascular smooth muscle cell normally contributes to the production of new interstitial collagen and maintenance of the extracellular matrix; however, this process is impaired in inflammatory states as being mediated by T cells [19]. Furthermore, matrix-degrading proteinases that are regulated by pro-inflammatory cytokines lead to a further loss of integrity of the fibrous cap. Pro-inflammatory cytokines lead to the expression of endothelial–leukocyte adhesion molecules that capture leukocytes such as the monocyte [20]. Within the plaque, activated macrophages also undergo the stress of the endoplasmic reticulum under these conditions. Over time, microvasculature also develops within the atherosclerotic plaque, and other molecules, such as the vascular cell adhesion molecule-1 (VCAM-1), have been implicated in driving this process [21].

After plaque rupture, a thrombus forms, which leads to the further accumulation of polymorphonuclear leukocytes and myeloperoxidases. This leads to further thrombus formation. In this setting of systemic inflammation, pro-inflammatory cytokines lead to the overproduction of IL-6. The acute phase response occurs with the production of fibrinogen, which can lead to thrombus formation, and plasminogen activator inhibitor-1, which can impair fibrinolysis.

Different steps of this inflammatory cascade have been targeted for the prevention and treatment of disease with limited success. However, several trials are ongoing, as detailed below. After presenting with acute myocardial infarction, patients with rheumatic immune-mediated inflammatory diseases are at high risk of recurrent myocardial infarction, as well as other outcomes such as mortality, heart failure, and coronary reintervention [22]. This is likely related to persistent inflammation and risk factors that led to their initial event. This residual inflammatory risk due to persistent inflammation should be assessed with a personalized approach to treatment as possible [23].

## 4. Acute Coronary Syndrome in Rheumatologic Disease

### 4.1. Rheumatoid Arthritis

Rheumatoid arthritis (RA) is the most common inflammatory arthritis, with a higher prevalence in industrialized and Western nations like the United States [24]. It has been shown to be associated with increased cardiovascular risk and mortality. Prior European Alliance of Associations for Rheumatology (EULAR) guidelines recommended a 1.5× multiplier to traditional risk model estimators in these patients [25]. Compared to the general population, cardiovascular disease incidence is increased by about 70% [26] and mortality is increased by 50% in patients with RA [27]. However, in a large, international cohort study, while much of the population’s attributable risk could be assigned to traditional risk factors, 30% was attributable to RA-specific characteristics such as seropositivity and the disease activity score [5].

Pro-inflammatory cytokines, such as TNF-a, IL-1, and IL-6, as well as T-cell activation, are activated in patients with RA. Patients with RA can have lower levels of cholesterol and LDL in what is known as the “lipid paradox” [28]. However, there are increased levels of oxidized LDL and decreased amount of HDL of which the atheroprotective effects are altered by chronic inflammation [29]. Certain cytokines, like IL-6 and TNF-α, lead to an increased uptake of LDL by the liver [30]. In rheumatoid arthritis specifically, LDL can become oxidized due to the inflammatory process [31]. These oxidized particles can cause endothelial cell damage through inflammation and can further promote TNF-α as well.

While glucocorticoids are often used to treat RA, especially during active flares, the long term use of glucocorticoids has been associated with a high risk of cardiovascular events, especially at higher doses (≥7.5 mg/day prednisone equivalent) [32]. However, this has not been seen with steroid-sparing agents, which have been shown to stabilize and decrease plaque by coronary computed tomography angiography and have been associated with lower risk of cardiovascular events [25,33,34]. Upon a diagnosis of RA, therapy with disease-modifying antirheumatic drugs (DMARDs) should be initiated [35]. There are a number of different DMARDs available for treatment, including conventional synthetic DMARDs (methotrexate, leflunomide, sulfasalazine, hydroxychloroquine), targeted synthetic DMARDs (baricitinib, filgotinib, tofacitinib, upadacitinib), and biological/biosimilar DMARDs (TNFi: adalimumab, certolizumab, etanercept, golimumab, infliximab; IL-6: sarilumab, tocilizumab; Co-stimulation-i: abatacept; CD20: rituximab). This regimen should be adjusted until a target of sustained remission or low disease activity is achieved.

Observational studies have shown an increased rate of ACS when patients with RA are not in remission, as well as an increased level of CRP [36,37]. Rheumatoid arthritis has been shown to be associated with a more severe presentation of acute coronary syndrome, with a higher incidence of ST Elevation Myocardial Infarction (STEMI), cardiac death, and in-hospital complications [38]. This association with in-hospital death can be seen in other studies as well [39,40]. Unfortunately, this association persists with time as rheumatoid arthritis has been associated with a higher risk of mortality, heart failure, and recurrent myocardial infarction at one year as well as lower survival, 49.2% vs. 82%, at five years compared to other patients presenting with STEMI [22,41]. Table 2 shows a summary of the findings of the studies on rheumatic diseases and acute coronary syndrome.

### 4.2. Psoriatic Arthritis

Psoriatic arthritis is a chronic inflammatory disease with diverse disease manifestations [75]. Per the Classification Criteria for Psoriatic Arthritis criteria, approximately 30% of patients with severe psoriasis, defined by nail and scalp involvement, develop psoriatic arthritis within ten years of diagnosis [76]. Traditional cardiovascular risk factors and cardiometabolic disorders are high in patients with psoriatic arthritis compared to the general population and are a leading cause of mortality [75,77]. Interestingly, patients without cardiovascular risk factors are often found to have subclinical atherosclerosis [78].

Cardiovascular disease in psoriatic arthritis is comparable to patients with rheumatoid arthritis and diabetes mellitus [75]. The mechanism is poorly understood like in most chronic inflammatory conditions; however, pro-inflammatory mediators, the upregulation of nitric oxide, and increased levels of the angiotensin-converting enzymes (ACEs) renin and endothelin-1 are postulated to increase endothelial dysfunction [79,80]. Some small studies in patients with moderate to severe psoriasis demonstrated a positive correlation between ACE levels and mean carotid intima–media thickness [79]. Increased ACE and renin levels not only increase traditional cardiovascular risk factors such as hypertension, but stimulate IL-6 release from endothelial cells, stimulating vascular smooth muscle proliferation [80].

In Polachek et al.’s meta-analysis of observational studies of 25,942 patients with psoriatic arthritis, the risk of myocardial infarction was 68% and the risk of developing incident myocardial infarction was 57% higher compared to the general population [81]. However, some studies have shown no difference in mortality following cardiovascular events in patients with psoriatic arthritis compared to the general population [41]. Furthermore, in their matched cohort study of 455 patients with psoriatic arthritis, Miyachi et al. revealed significantly lower 30-day in-hospital mortality (odds ratio [OR], 0.26; 95% confidence interval [CI], 0.08–0.85) [42]. This was postulated due to systemic inflammation suppression through biologic disease-modifying agents such as TNF-α inhibitors, interleukin-12/23 inhibitors, and interleukin-17 inhibitors. This is in alignment with other reviews and meta-analyses where systemic therapy was associated with a significant decrease in risk for all cardiovascular events in psoriatic arthritis or psoriasis [34]. In psoriasis, TNF-α inhibitors have been associated with an increased rate of lower incidences of myocardial infarction compared to other disease-modifying medications [82]. Overall, systemic therapy, with the exception of prolonged corticosteroid use, can reduce cardiovascular events by reducing disease activity and inflammation [75].

### 4.3. Systemic Lupus Erythematosus

Systemic lupus erythematous (SLE) is a multiorgan immune-mediated inflammatory disorder with wide clinical presentation. SLE is often associated with a bimodal pattern of mortality due to its early active disease state and later cardiovascular events, including myocardial infarction and stroke [83]. Compared to the general population, SLE patients have a two-fold higher risk of nonfatal cardiovascular events, and even in younger patients, the annual rates of cardiovascular events have increased, with a hazard ratio for myocardial infarction ranging from 2.6 to 5.1 [83]. Secondary antiphospholipid syndrome in SLE is an acquired pro-atherosclerotic and pro-thrombotic disease, which predisposes to premature CAD, thrombosis, and obstetric complications [84].

Lupus antibodies, such as anti-double-stranded DNA antibodies, are well known in the development of lupus nephritis and the progression to end-stage renal disease, which in itself increases cardiovascular risk [85]. Patients with positive and persistent anti-dsDNA antibodies were associated with endothelial dysfunction, proatherogenic dyslipidemia, accelerated atherosclerosis, and increased inflammatory mediators. These molecular processes associated with anti-dsDNA are partially due to alterations in molecular processes that drive immune and vascular activation [86]. IgG antibodies against HDL and apolipoprotein A-1 are increased in SLE and associated with the destabilization of atheromatous plaque [87]. Besides endothelial cell activation, SLE has been associated with increased neutrophil activation and neutrophil extracellular traps, which result in excess thrombosis, coagulation, and endothelial damage through oxidation, cytokine production, and citrullination [83].

While SLE is strongly associated with cardiovascular disease, studies have been inconsistent with the association between disease activity measurements and atherosclerosis [83]. Notably, carotid intima–media thickness and early evidence of plaque were predictive of future cardiovascular events [88]. Hydroxychloroquine is the mainstay treatment for glucocorticoid-sparing medication. It has been associated with a reduction in cholesterol, glycemic control, and reduced thrombosis [83,84]. Glucocorticoids are often used in active SLE flares, which are well known to increase blood pressure, blood glucose, cholesterol, and BMI. Recurrent and severe SLE is often treated with more advanced therapies such as immunomodulators/immunosuppressants or newer biologics, like belimumab and anifrolumab, especially to decrease glucocorticoid administration [89]. A significant impact on LDL and triglyceride levels was seen with a reduction in corticosteroids of greater than 5 mg/day [90,91]. Further direction is needed to address whether reducing systemic inflammation with disease-modifying medications, immunosuppressants, and newer novel biologics in SLE can improve abnormalities in coronary vascular function and cardiovascular outcomes [83,92].

### 4.4. Inflammatory Bowel Disease

Inflammatory bowel disease (IBD), consisting of ulcerative colitis (UC) and Crohn disease (CD), is characterized by systemic inflammation, periodic flares, and in some cases extra-gastrointestinal manifestations [59]. The prevalence of IBD in acute coronary syndrome (ACS) admissions has increased nearly 2-fold from 2005 to 2015 [58]. Multiple studies have demonstrated that IBD is associated with no difference in hospital mortality and survival [41,93]. However, during acute flares or persistent inflammation, IBD patients who presented with ACS tended to have worse outcomes compared to the general population, and these outcomes were comparable during quiescent activity. Notably, patients with IBD who presented with ACS had a lower prevalence of traditional cardiovascular disease risk factors, suggesting an unmeasured risk attributable to IBD-related inflammation [58].

Multiple etiologies have been proposed for the pathogenesis of atherosclerotic cardiovascular disease in patients with IBD [94]. IBD is associated with pro-inflammatory markers; TNF-alpha, IL-1 beta, and IL-6 are elevated in active disease and lead to the production of active oxygen species, increasing the risk of cardiovascular disease [95]. Gut microbiome abnormalities through local and systemic inflammation create barrier dysfunction and intestinal endothelial cell activation, initiating an inflammatory cascade, promoting procoagulant pathways, and altering glucose and lipid metabolism [94].

The prevalence of traditional modifiable risk factors in the IBD population prompts appropriate preventive and management strategies to reduce CVD risk [58]. The noninvasive biomarker fecal calprotectin provides information of intestinal inflammation by measuring fecal neutrophil, monocyte, and macrophage translocation to endothelial cells. CRP is also useful, though less sensitive [96]. IBD treatment consists of a stepwise approach with glucocorticoids, immunomodulators/immunosuppressants, and biologics. IBD patients treated with biologics, including TNF inhibitors, were associated with a 21% decreased risk of acute arterial events [97]. However, regression models have shown that disease activity, duration, and treatment do not significantly affect carotid intima–media thickness in patients with IBD [95].

### 4.5. Ankylosing Spondylitis

Ankylosing spondylitis (AS) is a chronic inflammatory disease that primarily affects the axial spine, entheses, and peripheral joints, and more frequently affects men [98]. AS is associated with cardiac conduction disorders, aortic regurgitation, and aortitis [60,99]. Chest pain is common in AS due to the disease involvement of the entheses of the thorax, but often presents with atypical symptoms [60]. AS is strongly associated with traditional cardiovascular factors such as diabetes mellitus, hypertension, dyslipidemia, obesity, and metabolic syndrome [100]. While some studies have shown a higher risk of myocardial infarction and stroke in patients with AS, others have shown no increased cardiovascular death or difference in long-term survival after myocardial infarction [41,60,101].

There is no clear mechanism for myocardial injury due to ankylosing spondylitis; however, similar to other chronic inflammatory disorders, active inflammation increases the risk of mortality [102]. It is suspected that accelerated atherosclerosis and endothelial dysfunction is driven by underlying inflammation [103]. In addition to an increased association with traditional cardiovascular risk factors, patients with AS may be at increased risk due to their medications used for management. Prior EULAR and ACR recommendations suggested prolonged, continuous disease management with NSAIDs over on-demand therapy [25,104]. Although NSAID use may increase cardiovascular risk, guidelines suggested reducing disease activity and inflammation may counterbalance this and decrease cardiovascular events. This was seen in a 8-year nationwide study with 22,929 patients where both NSAID and anti-TNF treatments in AS were associated with a lower risk of MACE occurrence [105]. Patients who are unable to tolerate or achieve symptom control with NSAID therapy are recommended TNF inhibitors prior to immunomodulators or immunosuppressants [25,104].

### 4.6. Gout

Gout is the most common inflammatory arthritis worldwide [63]. Here, inflammation is driven by the deposition of monosodium urate crystals due to hyperuricemia, and as with other chronic inflammatory disorders, this inflammation in gout is implicated in the development of atherosclerosis and subsequent presentation with myocardial infarction [62,66,106]. Gout was shown to have a significantly higher incidence of acute myocardial infarction, with an adjusted hazard ratio of 1.36 (95% CI, 1.04–2.76), although the risk was conditional on each patient’s cardiovascular risk profile [66]. A large prospective population-based cohort study in Sweden showed that patients with incident gout had a 44% increased risk of first-time ACS. This risk was higher in women, although it was also thought to be mainly driven by underlying comorbidities [63]. An increased short-term risk of major adverse cardiovascular events within 30 days of diagnosis has also been shown, as well as a higher likelihood of a cardiovascular event after a recent gout flare [61,64].

Elevated urate levels are well understood to trigger gout flares, with an increased deposition of monosodium urate crystals. However, the renal and gut excretion of urate is important in the regulation of serum urate. During acute gout flares, neutrophilic inflammation is due to the activation of the NLRP3 inflammasome [64,106]. This is associated with atherosclerotic plaque instability and rupture as well as an upregulated host response promoting oxidative stress and leading to plaque destabilization [106]. Underlying comorbidities are a major influence on cardiovascular events, as well as the gout-mediated oxidative stress of lipoproteins within atherosclerotic plaque.

Recent EULAR guidelines recommend the avoidance of medications that increase serum uric acid levels such as thiazide and loop diuretics in addition to the management of traditional risk factors, such as hypertension and cholesterol, as recommended by society guidelines [107]. While elevated serum urate levels greater than 8 mg/dL have a strong association with cardiovascular events, studies are conflicting on the efficacy of urate-lowering therapy, and currently, a cut-off value of less than 6 mg/dL is suggested to be beneficial in the reduction in cardiovascular events [107]. This could potentially be explained by a reduction in oxidative stress and improved endothelial function [63].

### 4.7. Systemic Sclerosis

Systemic sclerosis is a multiorgan disease associated with vascular injury, vascular and visceral fibrosis, and innate and adaptive immune activation with autoantibody production [108]. Systemic sclerosis has the highest mortality and morbidity among rheumatic diseases despite medical advancements due to its heterogenous organ involvement [109,110]. It is important to distinguish scleroderma, which refers to only cutaneous involvement from systemic sclerosis, which relates to cutaneous manifestations and other visceral involvement. Systemic sclerosis is associated with a high risk of mortality, heart failure, and recurrent myocardial infarction (adjusted HR 1.49, 95% CI: 1.40–1.58; adjusted HR 1.38, 95% CI: 1.27–1.5; adjusted HR 1.15, 95% CI: 1.05–1.27, respectively) [22]. In a meta-analysis with 14,813 study participants, patients with systemic sclerosis had elevated risk for cardiovascular disease (HR 2.36, 95% CI: 1.97–2.81) and myocardial infarction (HR 2.36, 95% CI: 1.71–3.25) [68]. Systemic sclerosis is known for microvascular disease, which is proposed to cause structural alterations and be predictive of macrovascular atherosclerosis; however, prior studies reported the prevalence of atherosclerosis to be similar to the general population [109]. Current EULAR recommendations on the treatment of systemic sclerosis highlight the need for further research on the efficacy of biological interventions on cardiovascular manifestations; notably, acute coronary syndrome is not listed as a clinical domain of presentation [110]. Due to its high heterogeneity in presence and the severity of skin and visceral involvement, it is not only difficult for clinical management, but also for trial design and concise interpretation in retrospective analyses.

### 4.8. Myositis

Inflammatory myopathy encompasses a spectrum of autoimmune myopathies with a broad spectrum of clinical presentations. These include polymyositis, dermatomyositis, immune-mediated necrotizing myopathy, anti-synthetase syndrome, and inclusion body myositis and are primarily characterized by immune-mediated muscle injury and varying levels of creatinine kinase elevation [111]. Patients with inflammatory myopathies have a higher prevalence of traditional risk factors and subclinical atherosclerosis [112]. Patients with polymyositis and dermatomyositis were shown to have an increased risk of myocardial infarction within the first year of diagnosis [113]. In a review of patients with ACS and immune-mediated inflammatory disease including 1127 patients with dermatomyositis, dermatomyositis was associated with a significantly elevated risk of mortality and recurrent myocardial infarction compared to the general population; (adjusted HR 1.29, 95% CI: 1.19–1.40 and adjusted HR 1.19, 95% CI: 1.06–1.33) [22]. Similarly, in an evaluation of the National Inpatient Sample, acute myocardial infarction and dermatomyositis were more likely to have worse in-hospital outcomes [114]. Furthermore, in an analysis of the NIH-sponsored All of Us dataset, patients with dermatomyositis or polymyositis were seen to have a reduced risk of cardiovascular events (adjusted HR 0.34; 95% CI 0.14–0.82; *p* = 0.02) when treated with non-steroidal immune-modulating therapies, including azathioprine, cyclophosphamide, hydroxychloroquine, intravenous immunoglobulin, methotrexate, mycophenolate mofetil/mycophenolic acid, and rituximab [69].

### 4.9. Mixed Connective Tissue Disease

Mixed connective tissue disease (MCTD) is an overlap syndrome that includes manifestations of multiple autoimmune diseases, such as SLE, systemic sclerosis, and autoimmune myositis. It is strongly associated with anti-U1-ribonucleoprotein antibodies [115]. Unfortunately, there are not many recent studies comparing cardiovascular risk in patients with MCTD, but it is well known to have multiple cardiac presentations with pericarditis, pericardial effusion, myocarditis, intimal hyperplasia, conduction disturbances, and small- and large-vessel vasculopathy [115]. These manifestations can be explained by the overlap syndrome with shared presentations as other rheumatic diseases. However, only a few case reports have described its association with myocardial infarction [116,117]. Intimal hyperplasia is suspected to occur due to the inflammatory-mediated proliferation of smooth muscle and extracellular matrices, and vasculopathy has been known to lead to pulmonary hypertension [118].

### 4.10. Sjögren Syndrome

Sjögren syndrome is a systemic autoimmune exocrinopathy affecting the salivary and lacrimal glands and commonly develops with other rheumatologic disorders, such as rheumatoid arthritis and SLE [119]. Elevated disease activity, including clinical manifestations such as Raynaud’s phenomenon, is associated with cardiovascular disease [120]. In an evaluation of the US Nationwide Inpatient Sample with 948 patients who presented with acute myocardial infarction and a diagnosis of Sjögren syndrome, Sjögren syndrome was associated with decreased odds of in-hospital mortality, cardiogenic shock, dysrhythmias, and prolonged length of stay [70]. However, an elevated EULAR Sjögren syndrome disease activity index (ESSDAI) ≥ 5 had an increased likelihood of myocardial infarction (OR 9.87; 95% CI: 1.17–83.49) [121]. This discrepancy from this nationwide retrospective study may be explained by the disease state and use of disease-modifying antirheumatic agents providing protective benefits by reducing systemic inflammation.

### 4.11. Antiphospholipid Syndrome

Antiphospholipid syndrome (APS) is a thrombo-inflammatory disease that is categorized as primary in the absence of other systemic autoimmune disorders or secondary, where it can be present in up to one-third of patients with SLE [122]. Antiphospholipid antibodies are detected during multiple settings such as rheumatic disease, obstetric morbidity, thrombocytopenia, livedo reticularis, false positive syphilis screening, and prolonged activated partial thromboplastin time. Patients with persistent antiphospholipid antibodies are associated with cardiovascular risk factors and are used for thrombosis risk prediction tools [122]. APS leads to the inappropriate activation of hemostatic factors in addition to immune-mediated thrombosis through the activation of the complement pathway, monocytes, and neutrophils [122]. An analysis of the National Inpatient Sample including 1180 patients with antiphospholipid syndrome presenting with ACS showed a strong correlation between the disease and presentation with ACS (OR 2.18; 95% CI: 1.55–3.09) independent of traditional risk factors. However, this elevated risk was only before the age of 40 [49].

## 5. MINOCA

Myocardial infarction with non-obstructive coronary arteries (MINOCA) can present similarly to other acute coronary syndromes. These patients have a lower burden of atherosclerosis as well as a lower frequency of typical risk factors and comorbidities. While these patients make up a small portion of all patients presenting with myocardial infarction, they include a wide range of etiologies. Typically, these can be divided into epicardial and microvascular causes [123]. Epicardial causes include plaque rupture, plaque erosion, coronary spasm, and spontaneous coronary artery dissection. The microvascular causes include spasm, coronary thromboembolism, and coronary microvascular dysfunction [124]. However, a wide differential, including causes of Type II MI, should be considered. Frequently, these patients are younger, are commonly women, and have a higher incidence of autoimmune disease. Further work-up and management are important since long term mortality and rates of major adverse cardiovascular events are as high with MINOCA as in myocardial infarction with obstructive CAD [125].

Autoimmune disorders have also been associated with an increased risk of MINOCA. This is thought to be due to systemic inflammation and its effects on endothelial dysfunction [126]. Endothelial dysfunction can lead to microvascular dysfunction; however, hypercoagulability and microvascular spasm are also possible contributors. MINOCA has been described in several different autoimmune disorders, including antiphospholipid syndrome and SLE [127,128].

Coronary microvascular dysfunction (CMD) is a subset of MINOCA in which there are abnormalities in the function of the microvascular flow that can lead to ischemia. This dysfunction is commonly related to endothelial dysfunction as well as the function of vascular smooth muscle cells. It has a higher prevalence in women and can frequently lead to misdiagnosis or additional, unnecessary testing [129]. Inflammatory conditions such as systemic lupus erythematosus (SLE) and rheumatoid arthritis are associated with CMD [130]. When assessed with positron emission tomography–computed tomography, patients with SLE were shown to have a higher prevalence of CMD and a more severe reduction in their myocardial flow reserve [92]. In patients presenting with angina and acute coronary syndrome who do not have evidence of coronary artery obstruction, it is important to consider CMD and other types of MINOCA, as diagnosis can lead to alternative management.

## 6. Treatment

In patients with rheumatologic disease presenting with acute coronary syndrome, the management approach should not differ drastically from that for typical patients. While the most recent update of the ESC guidelines does not have any treatment recommendations for such patients, it does for the first time include a comment on anti-inflammatory drugs [131]. The guidelines give a IIb recommendation for the consideration of the use of low-dose colchicine if other risk factors are insufficiently controlled or if recurrent cardiovascular disease events occur under optimal therapy. While the inflammatory process has been a targeted pathway of treatment in acute coronary syndrome, limited success has been made thus far. Table 3 provides a summary of recent randomized controlled trials targeting the treatment of inflammation.

In the Colchicine Cardiovascular Outcomes Trial (COLCOT), 4745 patients with a recent ACS event were enrolled and shown to have a significant reduction in a composite endpoint of cardiovascular death, resuscitated cardiac arrest, myocardial infarction, stroke, or urgent revascularization when treated with low-dose colchicine (0.5 mg daily) as compared to a placebo [132]. While not in the acute setting, the Low-dose Colchicine trial-2 (LoDoCo2) enrolled 5522 patients with chronic coronary syndrome and showed a significantly lower similar composite endpoint of cardiovascular death, myocardial infarction, stroke, or ischemia driven revascularization [133]. These trial results have led to the recommendation of colchicine use, while not in the acute setting, for further risk mitigation after the control of other risk factors. More recently, after the publication of the most recent guidelines, several trials have looked at the use of colchicine in the acute myocardial infarction setting. The CLEAR trial looked at 7062 randomized patients with myocardial infarction to receive either colchicine or placebo and did not show a difference in the primary efficacy outcome (9.1 vs. 9.3%, CI 0.85–1.16, *p* = 0.093) at three years of a composite of death from cardiovascular causes, recurrent myocardial infarction, stroke, or unplanned ischemia-driven coronary revascularization [134]. In a subgroup of the studied patients, there was no difference in their C-reactive protein levels measured at three months (−1.2 mg, 95% CI, −1.81 to −0.75), although there was a higher percentage of patients with diarrhea (10.2 vs. 6.6%, *p* < 0.001). Thus, in the largest trial performed to date, colchicine does not seem to provide long-term benefits to patients presenting with myocardial infarction.

**Table 3:** Summary of randomized trials targeting treatment of inflammation.

Study	Year	*n*	Intervention	Population	Primary Endpoint
LoDoCo [135]	2013	532	Colchicine 0.5 mg daily	Stable Coronary Disease	Lower composite of acute coronary syndrome, out-of-hospital cardiac arrest, or noncardioembolic ischemic stroke (5.3 vs. 16% HR 0.33; CI 0.18–0.59; *p* < 0.001)
COLCOT [132]	2019	4745	Colchicine 0.5 mg daily	Within 30d of MI	Lower composite of death from cardiovascular causes, resuscitated cardiac arrest, myocardial infarction, stroke, or urgent hospitalization for angina leading to coronary revascularization (5.5 vs. 7.1%, CI 0.61–0.96; *p* = 0.02)
LoDoCo-MI [136]	2019	237	Colchicine 0.5 mg daily	Acute MI	No difference in CRP level ≥ 2 mg/L after 30 days of treatment (44 vs. 50%, *p* = 0.35)
LoDoCo2 [133]	2020	5522	Colchicine 0.5 mg daily	Chronic Coronary Disease	Lower composite of cardiovascular death, spontaneous myocardial infarction, ischemic stroke, or ischemia-driven coronary revascularization (6.8 vs. 9.6%, CI 0.57–0.83; *p* < 0.001)
COPS [137]	2020	795	Colchicine 0.5 BID for one month, 0.5 mg daily for 11 months	ACS	No difference in composite of all-cause mortality, ACS, ischemia-driven unplanned urgent revascularization, and noncardioembolic ischemic stroke (24 vs. 38 events; *p* = 0.09). Higher rate of total death (8 vs. 1; *p* = 0.017)
CLEAR [134]	2024	7062	Colchicine	Acute MI	No difference in composite of death from cardiovascular causes, recurrent myocardial infarction, stroke, or unplanned ischemia-driven coronary revascularization (9.1 vs. 9.3%, CI 0.85–1.16, *p* = 0.093)
TIPTOP [138]	2014	110	Doxycycline 100 mg BID for 7d	STEMI with LVEF < 40%	Reduced increase in LVEDVi at 6 months (0.4% vs. 13.4%; *p* = 0.012)
LATITUDE-TIMI 60 [139]	2016	3503	Losmapimod (p38 Mitogen-activated protein kinase)	Acute MI	No difference in composite of cardiovascular death, MI, or severe recurrent ischemia requiring urgent coronary revascularization (8.1 vs. 7.0%, HR 1.16; CI 0.91–1.47; *p* = 0.24)
EMBRACE-STEMI [140]	2016	118	MTP-131	First-time anterior STEMI	No decrease in infarct size as assessed by CK-MB AUC (5570 ± 486 ng h/mL 5785 ± 426 ng h/mL; *p* = NS)
CANTOS [141]	2017	10,061	Canakinumab 150 mg every three months	Prior myocardial infarction and hsCRP ≥ 2 mg/L	Lower composite of nonfatal myocardial infarction, nonfatal stroke, or cardiovascular death (HR 0.85, CI 0.74–0.98; *p* = 0.021)
CIRT [142]	2018	4786	Methotrexate 15–20 mg weekly	Prior myocardial infarction or multivessel coronary disease with T2DM or metabolic syndrome	No difference in composite of nonfatal myocardial infarction, nonfatal stroke, or cardiovascular death (3.46 vs. 3.43 per 100 person-years; HR 1.01; CI 0.82–1.25)
MRC-ILA [143]	2015	182	Daily subcutaneous IL-1receptor agonist for two weeks	NSTE-ACS	Lower AUC CRP (22 mg d/L vs. 43.5 mg d/L, *p* = 0.0028)
VCUART3 [144]	2020	99	Anakinra 100 mg once or twice daily	STEMI	Lower hsCRP area under the curve (67 vs. 214; *p* < 0.001)
STAT-MI [145]	2018	28	Tocilizumab	Acute MI	No difference in MACE at 30 days
ASSAIL MI [146]	2021	199	Tocilizumab	STEMI	Increased myocardial salvage index as measured by magnetic resonance imaging after 3 to 7 days (adjusted between-group difference 5.6 (CI 0.2–11.3), *p* = 0.04)
RITA-MI [147]	2022	24	Rituximab	STEMI	Appears well tolerated; decrease in circulating B-cells
CLEVER-ACS [148]	2022	150	Everolimus	STEMI	No difference in myocardial infarct size by CMR (−14.2 g vs. −12.3 g, *p* = 0.99)
Goflikicept [149]	2024	102	Goflikicept	STEMI	Reduction in AUC of hsCRP at 14 days
PULSE-MI [150]	2024	530	Methylprednisolone 250 mg prehospital	STEMI	No difference in final infarct size after size (5% vs. 6%, *p* = 0.24)

The targeted treatment of inflammation in acute myocardial infarction started with the CANTOS (the Canakinumab Anti-inflammatory Thrombosis Outcomes Study) trial, which randomized over 10,000 patients with prior myocardial infarction and elevated CRP levels to treatment with a therapeutic monoclonal antibody, canakinumab, targeting interleukin-1β, versus with a placebo, and showed a significantly lower rate of recurrent cardiovascular events with treatment [141]. This finding was significant at a dose of 150 mg every three months (HR 0.83, 95% CI, 0.73 to 0.95; *p* = 0.005) and had a 37% reduction in CRP levels, even though patients did not have a significant difference in LDL levels. This was the first major trial to show a benefit of non-targeted inflammatory treatment in patients with myocardial infarction. However, in the CIRT (Cardiovascular Inflammation Reduction Trial), low-dose methotrexate or a matching placebo was used in 4786 patients with prior myocardial infarction or multivessel coronary disease who also had type 2 diabetes or metabolic syndrome. The methotrexate did not result in lower interleukin-1β, interleukin-6, or C-reactive protein levels or a reduction in the primary endpoint of a composite of nonfatal myocardial infarction, nonfatal stroke, or cardiovascular death (3.46 vs. 3.43 per 100 person-years; HR, 101; 95% CI, 0.82 to 1.25) [142]. The ASSAIL MI (assessing the effect of Anti-IL-6 treatment in myocardial infarction) trial was conducted to test the hypothesis that the administration of tocilizumab would increase the myocardial salvage index, as measured by cardiac magnetic resonance imaging, in patients presenting with acute STEMI. A total of 101 patients were randomized to receive a single infusion of 280 mg tocilizumab and 98 to receive a placebo. While the myocardial salvage index was larger and microvascular obstruction was less extensive in the tocilizumab group, there was no significant difference in infarct size, and adverse events were evenly distributed across the treatment groups [146]. In the LATITUDE-TIMI 60 study, 1738 patients were randomized to losmapimod, a p38 Mitogen-activated protein kinase, and 1765 to a placebo, but the use of losmapimod did not reduce the risk of major ischemic cardiovascular events at twelve weeks [139]. In a small trial, VCUART3 (Virginia Commonwealth University Anakinra Remodeling Trial 3), an IL-1 blockade with anakinra significantly reduced the systemic inflammatory response compared with the placebo, as measured by CRP [144]. The MRC-ILA Heart Study had previously shown similar findings of a reduction in CRP levels, and MACE levels were similar at one and three months but higher at one year in the IL-1ra group [143]. Other treatment targets, including IL-18 (GSK1070806) [151] and NLRP3 inflammasome-targeted therapy (VX-765, VX-740, Arglabin) [152,153], have been studied for the reduction in inflammation and development of atherosclerosis; however, these have yet to be studied for acute coronary syndrome.

Other, non-targeted therapies are still undergoing evaluation, as outlined in Table 4. ARTEMIS (Effects of Ziltivekimab versus Placebo on Cardiovascular Outcomes in Patients With Acute Myocardial Infarction), a large-scale trial of approximately 10,000 patients, recently launched to test whether a novel interleukin-6 inhibitor (Ziltivekimab; Novo Nordisk, Bagsværd, Denmark) could reduce the risk of recurrent events in the setting of acute MI (NCT06118281). Several smaller trials, including Doxycycline in STEMI (NCT03508232) to assess matrix metalloproteinase-2 inhibition and IVORY (Low-Dose Interleukin-2 for the Reduction in Vascular Inflammation in Acute Coronary Syndromes) (NCT04241601) to test aldesleukin (recombinant IL-2), use imaging findings as a primary outcome, such as CMR or FDG-PET/CT.

The inflammatory response after ACS is complex. While several studies have shown early benefits, these have been unable to translate into meaningful clinical outcomes. While canakinumab was shown to have a significant reduction in MACE, it was not approved for use by the FDA for its applied indication. Ongoing trials are set to target IL-6, IL-2, MMP, and CD20. However, only ARTEMIS includes a primary outcome of cardiovascular death, nonfatal myocardial infarction, and nonfatal stroke after acute MI. Prior attempts at addressing IL-6 with tocilizumab did not show any clinical benefit. The interleukins as well as the NLRP3 inflammasome seem to be promising targets for intervention. It is possible that individually targeted treatment is needed, and thus widespread clinical benefit has not been borne out in studies so far. It is also possible that the timing of the administration of the drugs is not ideal or that the correct target along the inflammatory pathway has not been selected. Future studies should hope to refine these questions.

Patients with rheumatologic disease presenting with acute coronary syndrome have been shown to have more severe presentations as well as a higher incidence of cardiac death and in-hospital complications [38,39,40]. However, this has not been borne out in observational data of all rheumatologic diseases, such as psoriatic arthritis or IBD [42,93]. Nevertheless, due to their special characteristics, it is important to have a high index of suspicion and careful attention to patient risk factors. In the acute setting, management for ACS should not be different than for patients without rheumatologic diseases [131,154].

Patients presenting with symptoms consistent with acute coronary syndrome should undergo work-up, including vital signs, a physical exam, an electrocardiogram, and a laboratory evaluation including biomarkers such as troponin. Patients with a rheumatologic disease might present with atypical symptoms or could potentially be diagnosed with an alternate etiology for their pain. Further work-up might include echocardiograms, non-invasive imaging, or coronary angiography for appropriate candidates.

Antithrombotic therapy is an essential part of management for acute coronary syndrome. Similar anticoagulation and dual antiplatelet therapy and duration should be given for patients with rheumatologic diseases presenting with ACS. While some rheumatologic diseases have been associated with higher bleeding risk, this can perhaps be attributed to the use of NSAIDs and glucocorticoids. Caution should be taken, and mitigation strategies, such as the use of proton pump inhibitors in patients at higher-than-average risk of gastrointestinal bleeds, should be considered when necessary.

It is important to periodically assess for drug–drug interactions. Statins in combination with colchicine have been shown to have increased exposure and increased risk for muscle-related toxicity. For this reason, closer monitoring is recommended but medications can still be used together [155]. Cyclosporine, Tacrolimus, Everolimus, and Sirolimus are rarely used but also could be potentially harmful in combination with lovastatin, simvastatin, and pitavastatin, and if used with >10 mg/d of atorvastatin should be closely monitored for signs of muscle-related toxicity [155].

The coadministration of colchicine and rosuvastatin, fluvastatin, lovastatin, pitavastatin, and pravastatin is reasonable when clinically indicated. Dose reductions may be considered for atorvastatin, simvastatin, and lovastatin, given the potential for interactions mediated by both the CYP3A4 and permeability glycoprotein (P-gp) pathways. The P glycoprotein plays an important role in the intestinal efflux of clopidogrel and ticagrelor and thus can be affected by inhibitors (cyclosporine) or inducers of this transporter. Different cytochrome P450 enzymes are important in the metabolism of clopidogrel (2C19, 3A4), prasugrel (3A4, 2B6), and ticagrelor (3A). These, of course, can be influenced by other medications that act on this pathway. Most common would be inhibitors of the 3A4 pathway; however, they are not too frequently used in the treatment of rheumatologic diseases [156]. It is possible that an increased systemic inflammatory state can reduce the antiplatelet effect; however, more studies are needed [157].

## 7. Prevention

Prevention is arguably the most important part of the treatment of acute coronary syndrome in patients with rheumatologic diseases. The European League against Rheumatism (EULAR) provides recommendations for cardiovascular risk management in rheumatic and musculoskeletal diseases, including systemic lupus erythematosus and antiphospholipid syndrome [107]. In these patients, it is important to assess traditional cardiovascular risk factors. These might occur at a higher incidence in this particular patient population.

For many common diseases, treatment can parallel recommendations used for the general population. Occasionally, there are specific differences that occur. For blood pressure management, diuretics should be avoided in gout, as well as beta blockers in patients with systemic sclerosis. A blood pressure target <130/80 mm Hg should be considered in patients with lupus as lower blood pressure has been associated with lower cardiovascular events. For those with lupus nephritis and a urine protein-to-creatinine ratio >500 mg/g, an angiotensin-converting enzyme inhibitor or angiotensin receptor blocker can be preferentially used.

Lipid management should follow recommendations used in the general population. As before, statins can occasionally be associated with drug–drug interactions, so a review should be conducted with each new prescription of a medication. However, the potential interaction of myotoxicity with statin treatment and colchicine is rare. Occasionally, some patients with rheumatoid arthritis do have lower LDL cholesterol levels in what is known as the “lipid paradox”; however, treatment should be pursued as indicated based on their ASCVD risk.

In the absence of coronary artery disease or another indication, low-dose ASA for primary prevention is not recommended. However, for aPL carriers with a high-risk profile with or without traditional risk factors, low-dose ASA is recommended [107]. In patients on antiplatelet or anticoagulant therapy, consideration should be given to concomitant therapy with NSAIDs and glucocorticoids, keeping in mind elevated bleeding risk.

With the increasing complexity of treatment options available for these patients, in addition to their various comorbidities, specialty management is essential. For prevention, patients with rheumatic disease require enhanced cardiovascular risk stratification and screening for comorbidities. In addition to the management of the usual modifiable cardiovascular risk factors, underlying inflammation and disease activity has been shown to correlate with the risk of cardiovascular events. Thus, the treatment of underlying disease and inflammation is of paramount importance. However, the lowest possible glucocorticoid use should be targeted. This may often mean using biologics or disease-modifying antirheumatic drugs. Due to the complex nature of these patients, specific clinics within the field of cardio-rheumatology have been suggested [158,159]. Both the cardiologist and the rheumatologist have the disease and pharmacologic expertise required for management. Further collaboration will only become more important in the future as more targeted therapies are developed.

## 8. Conclusions

Despite advancements in treatment and better recognition, cardiovascular risk remains elevated in patients with rheumatologic diseases. This is a source of significant morbidity and mortality in these patients. While the management of patients with rheumatic diseases who present with acute coronary syndrome is not dissimilar to the general population, these patients can frequently have worse in-hospital and long-term outcomes. After focusing on their acute clinical management, consideration should be given to further risk reduction and the prevention of future episodes. In addition to the management of cardiovascular risk factors, the treatment of underlying inflammation through the management of ongoing disease activity is of paramount importance. Special attention must be paid to medications and potential interactions. Disease management is complex, and multidisciplinary care between rheumatology, cardiology, and primary care providers should be used to provide the best care for patients.

While inflammation plays a role in the development of atherosclerosis as well as the acute manifestation of this disease, treatment focused on inflammation has yet to provide much of a clinically meaningful effect. Ongoing trials continue to look at potential areas of treatment, and new targets in the inflammatory cascade, such the inflammasome in addition to specific interleukins, might provide therapeutic benefit. In the future, targeted precision medicine might be able to give an individual approach to each patient. If available, this might lead to individualized approaches for the treatment of inflammation in acute coronary syndrome for each rheumatologic disease. For now, we must continue to focus on the reduction in overall disease activity and aggressive risk factor management, as well as preventive care.

## Figures and Tables

**Table 1:** Pathophysiology of rheumatic diseases and mechanism of coronary injury.

Disease	Mechanism of Coronary Injury
Rheumatoid arthritis	Upregulation of pro-inflammatory cytokines, including TNF-a, IL-1, IL-6, and T-cell activation.
Psoriatic arthritis	No precise correlation; however, inflammatory mediators postulated with interleukins (IL)-1,4,6,8, and 12 and tumor necrosis factor-a (TNF-a), the upregulation of IL-17 and NO, and an association with higher levels of angiotensin-converting enzymes (ACEs), renin and endothelin-1. Disease-modifying medications associated with cardiovascular disease.
Systemic lupus erythematous (SLE)	Accelerated atherosclerosis due to SLE-driven inflammation, facilitated by steroids, and antiphospholipid antibodies.
Inflammatory bowel disease (IBD)	Multifactorial due to chronic inflammation, gut microbiome abnormalities, endothelial dysfunction, thrombosis, lipid dysfunction, and effects of corticosteroid usage during disease flares.
Ankylosing spondylitis	Accelerated atherosclerosis and endothelial dysfunction in the background of inflammation.
Gout	Elevated uric acid levels increase risk of cardiovascular disease; no clear molecular or genetic link between gout and cardiovascular disease.
Systemic sclerosis	Chronic inflammation with accelerated atherosclerosis and endothelial dysfunction
Myositis/Dermatomyositis	Chronic inflammatory condition and increased risk of traditional cardiovascular risk factors.
Mixed connective tissue disease (MCTD)	Chronic inflammatory condition with elevated risk of carotid intima–media thickness.
Sjogren’s disease	Chronic inflammatory condition with increased risk of cardiovascular disease with presence of Raynaud’s phenomenon.
Antiphospholipid syndrome (APLS)	Associated with higher prevalence of microvascular disease and atherosclerosis.
Chronic microvascular disease (CMD)	Chronic inflammation with endothelial and vascular smooth muscle cell dysfunction.

**Table 2:** Summary of studies on acute coronary syndrome in rheumatic diseases.

Disease	First Author	Year	*n*	Study Type (Location)	Findings	Study Limitations
**Rheumatoid Arthritis**	Delcoigne [36]	2023	41,250	Observational Cohort (Sweden, Norway)	Active disease associated with increased risk of developing ACS event (adjusted HR 1.52, 95% CI: 1.24–1.48)	Lacks information on potential confounders such as the dose of corticosteroid and concomitant NSAID or COX-II inhibitors.
	Boukhris [41]	2022	20	Prospective Registry (Italy)	Five-year survival lower in RA patients (49.2% vs. 82%; *p* = 0.001)	Sample size of patients is relatively limited.
	Wassif [22]	2022	46,747	Retrospective Cohort (US-CMS)	RA associated with higher risk of mortality, HF, and recurrent MI at 1-year (adjusted HR 1.13, 95% CI: 1.12–1.15; adjusted HR 1.12, 95% CI: 1.1–1.14; adjusted HR 1.06, 95% CI: 1.04–1.08)	Limited to Medicare patients; may not be generalizable to younger patients. Lacks information on duration, disease-specific activity, laboratory and imaging data, and medications.
	Lai [39]	2020	748	Population Study (Taiwan)	RA associated with in-hospital death (OR: 1.61, 95% CI: 1.33–1.95) and overall death. RA associated with increased risk of MACE (HR: 1.28, 95% CI: 1.18–1.38)	Database lacks extent and distribution of CAD, exact timing of reperfusion procedures after AMI, smoking status, and use of immunomodulators. Taiwan has reimbursement reduction, which may restrict medical expenditures.
	Elbadawi [40]	2020	123,783	Retrospective Cohort (US–NIS)	RA independently associated with lower rates of in-hospital mortality (adjusted OR 0.90; 95% CI, 0.81–0.99; *p* = 0.03)	Lacks data on medications, immune-target therapies, and laboratory and imaging data. Possibility for selection bias. Unmeasured confounders.
	Mantel [38]	2015	1135	Nationwide Population-Based Cohort Study (Sweden)	Higher incidence of sudden cardiac death, STEMI, and in-hospital complications in RA patients. Higher rate of 30-day death (HR: 1.57, 95% CI: 1.30–1.89)	Limited information on smoking and BMI. Data from RIKS-HIA used to assess hospital ACS interventions. Lower coverage of patients over 80 years of age.
**Psoriatic Arthritis**	Wassif [22]	2022	3098	Retrospective Cohort (US–CMS)	Psoriasis associated with lower risk of mortality at 1 year (adjusted HR 0.92, 95% CI: 0.87–0.98)	Limited to Medicare patients; may not be generalizable to younger patients. Lacks information on duration, disease-specific activity, laboratory and imaging data, and medications.
	Miyachi [42]	2022	455	Nationwide Retrospective Cohort Study (Japan)	Psoriasis associated with decreased 30-day in-hospital mortality (odds ratio [OR], 0.26; 95% confidence interval [CI], 0.08–0.85)	Unable to account for psoriasis severity, use of biologics, and discharged patients who died within 30 days of admission.
	Boukhris [41]	2022	18	Prospective Registry (Italy)	No significant difference in 5-year survival between patients with and without psoriasis (92.9% vs. 81.5%; *p* = 0.46)	Sample size of patients is relatively limited. Unable to assess a corticosteroid dose–effect response. Unable to exclude information bias during clinical history collection.
	Karbach [43]	2020	9028	Nationwide Inpatient Sample (Germany)	Psoriasis associated with lower in-hospital death rate (7.1% vs. 12.4%; *p* < 0.001)	Results are limited based on search strategy using ICD-10 and OPS codes, which may lead to underreporting and undercoding. No exact classification of disease severity is given.
	Desai [44]	2018	658	Retrospective Cohort (US–NIS)	Overall mortality was lower in Psoriasis group (3.3% vs. 5%) (*p* < 0.001)	Results are limited based on search strategy using ICD-9 CM diagnostic code 410.
	Ahlehoff [45]	2011	462	Population Cohort Study (Denmark)	No increased risk of all-cause death (HR: 1.18, 95% CI: 0.97–1.43), but increased risk of MACE (HR: 1.26, 95% CI: 1.04–1.54) in case of psoriasis	Cohorts based on prescription history for topical vitamin D derivatives. Predominantly Caucasian population. Unable to account for confounders such as smoking and BMI.
**Systemic Lupus Erythematous**	Abdul Jabbar [46]	2024	16,790	Retrospective Cohort (NIS–US)	In-hospital mortality, length of stay, and total cost were statistically similar in the presence or absence of SLE	Limited to NIS data and unable to consider observation-only hospital stays; may underrepresent certain conditions and procedures.
	Bello [47]	2023	62,875	Meta-Analysis	SLE had statistically significantly higher RRs (95% CIs) of MI (2.92 [2.45–3.48]) and CVD (2.24 [1.94–2.59])	Inclusion of multiple study types likely contributed to the heterogeneity observed for most endpoints. Unable to examine some factors affecting CVD in SLE, such as smoking and dyslipidemia.
	Wassif [22]	2022	7362	Retrospective Cohort (US–CMS)	SLE associated with high risk of mortality, HF, recurrent MI, and stroke (adjusted HR 1.23, 95% CI: 1.19–1.27; adjusted HR 1.24, 95% CI 1.19–1.30; adjusted HR 1.18, 95% CI: 1.13–1.24; adjusted HR 1.11, 95%: 1.13–1.24)	Limited to Medicare patients; may not be generalizable to younger patients. Lacks information on duration, disease-specific activity, laboratory and imaging data, and medications.
	Sagheer [48]	2022	2500	Retrospective Cohort (US–NRD)	Patients with SLE had higher 30-day readmission rate (15.5% vs. 12.5%; adjusted OR: 1.33, CI: 1.12–1.5), and in-hospital death (OR: 1.40, 95% CI: 1.1–1.79)	Limited to inpatient hospitalizations. Relied on ICD-10-CM/PCS codes. Lacks information on medications, laboratory values, and detailed clinical history.
	Whittier [49]	2022	8575	Retrospective Cohort (US-NIS)	Multivariate analysis of 18–40-year-old SLE strongly associated with ACS hospitalizations (odds ratio, 2.18; 95% confidence interval, 1.814–2.625)	Database relies on billing codes and lacks information on severity, duration of risk factors, or SLE disease activity.
	Yafsova [50]	2021	3411	Nationwide Cohort (Denmark)	Absolute 10-year risks of outcomes for myocardial infarction: 2.17% (95% CI: 1.66% to 2.80%) in SLE patients	Possible confounders cannot be excluded. Predominantly Caucasian population. Lacks data on covariates.
	Lai [39]	2020	256	Nationwide Cohort (Taiwan)	SLE associated with in-hospital death (OR: 2.31, 95% CI: 1.62–3.28). SLE associated with increased risk of MACE (HR: 1.46, 95% CI: 1.27–1.69)	Database lacks extent and distribution of CAD, exact timing of reperfusion procedures after AMI, smoking status, and use of immunomodulators. Taiwan has reimbursement reduction, which may restrict medical expenditures.
	Ando [51]	2019	4666	Retrospective Cohort (NIS–US)	In-hospital death (9.1% vs. 11.8%; OR 0.75; *p* = 0.07) and MACE similar between SLE and no SLE patients with STEMI and with NSTEMI (4.1% vs. 4.5%, OR 0.90; *p* = 0.51)	Database relies on billing codes and lacks information on severity or duration of risk factors or SLE disease activity and severity of CAD; possible confounders not accounted for.
	Ke [52]	2019	151	National Cohort (Taiwan)	In-hospital death higher in SLE (OR: 1.98, 95% CI: 1.2–3.26)	Lacks predictors of post-MI mortality, disease duration, and treatment prior to AMI.
	Lin [53]	2014	1207	Nationwide Retrospective Cohort (Taiwan)	Post-AMI death higher in SLE (OR: 2.60, 95% CI: 1.09–6.19)	Lacks data on major organ involvement or severity of SLE, laboratory data, severity of AMI, and treatments. Limited to adults aged older than 20 years with new-onset SLE only.
	Shah [54]	2009	2192	Retrospective Nationwide Cohort (US–NIS)	In-hospital death higher in SLE (HR: 1.65, 95% CI: 1.33–2.04)	Possible unaccounted confounders and exclusion of inactive or mild SLE.
**Inflammatory Bowel Disease**	Nasir [55]	2022	951	Retrospective Nationwide Cohort (US-NHIS)	IBD associated with increased odds of having ASCVD (OR 1.58, 95% CI 1.17–2.13)	Does not include IBD subtypes. Lacks IBD severity and duration, as well as medications used for both IBD and ASCVD.
	Popovic [56]	2022	1470	Retrospective Nationwide Cohort (France)	No differences in all-cause death (9% vs. 8.3, *p* = 0.729), stroke (0.8% vs. 0.6%, *p* = 0.656), hospitalization for heart failure (3.3% vs. 3.5%, *p* = 0.846), or recurrent myocardial infarction (2.9% vs. 1.9%, *p* = 0.33) between IBD and no-IBD patients	Retrospective, single-system database
	Sinh [57]	2021	7391	Retrospective Nationwide Cohort (US–NIS)	No difference in hospital mortality in patients with or without ulcerative colitis (7.75% vs. 7.05%; *p* = 0.25) or with or without Crohn’s disease (6.50% vs. 6.59%; *p* = 0.87)	Database relies on billing codes and lacks information on severity or duration of risk factors or IBD activity.
	Pemmasani [58]	2020	24,220	Retrospective Nationwide Cohort (US–NIS)	In-hospital mortality rate lower in patients with vs. without IBD (3.9% vs. 5.3%; OR: 0.81; 95% CI: 0.69–0.96)	Data, based on hospitalizations, rely on correct coding of diagnostic and procedure codes. Lacks information on IBD severity, medication use for IBD or ACS, and angiographic data.
	Kristensen [59]	2014	1030	Retrospective Nationwide Cohort (Denmark)	IBD associated with recurrent myocardial infarction (HR: 1.21, 95% CI: 0.99–1.49), all-cause death (HR: 1.14, 95% CI: 1.01–1.28), and composite outcome of recurrent myocardial infarction, cardiovascular death, or stroke (HR: 1.17, 95% CI: 1.03–1.34)	Lacks clinical information, including anatomic localization, extent of CAD, and use of anticoagulants during the MI hospitalization. Estimation of IBD activity was based on medical treatment and admissions. Flare duration of 120 days was arbitrary defined.
**Ankylosing spondylitis**	Boukhris [41]	2022	15	Prospective Registry (Italy)	No difference in 5-year survival between AS and no AS (100% vs. 81.5%; *p* = 0.29)	Sample size of patients is relatively limited. Unable to assess a corticosteroid dose–effect response. Unable to exclude information bias during clinical history collection.
	Södergren [60]	2020	292	Retrospective Nationwide Cohort (Sweden)	No difference in cardiovascular death, but overall death (days 31–365) increased among patients with AS vs. comparators (HR: 2.0, 95% CI: 1.3–3.0)	Lower coverage of patients over 80 years of age. Proportion that died from AMI or SCD prior to hospitalization is not known.
**Gout**	Cipolletta [61]	2024	4398	Retrospective Nationwide Cohort (UK)	Incidence of cardiovascular events significantly higher in the first 30 days after first gout diagnosis (31.2 [95% CI 27.1–35.9])	Possible unmeasured confounders. Gout severity based on hospitalization.
	Sedighi [62]	2024	132,000	Retrospective Nationwide Cohort (Germany)	Association between gout and subsequent cardiovascular diseases of angina pectoris (HR 1.61; 95% CI 1.49–1.74) and myocardial infarction (HR 1.36; 95% CI 1.23–1.50)	Data subjected to misclassification and undercoding. Lacks disease severity.
	Drivelegka [63]	2022	20,146	Prospective Nationwide Cohort (Sweden)	Gout cohort with increased risk for first-time ACS event (HR 1.44 [95% CI 1.33–1.56]), and higher in women (HR 1.64 [95% CI 1.41–1.90])	Possible misclassification bias and underestimation based on ICD codes. Not able to adjust for factors related to gout severity, including urate levels.
	Cipolletta [64]	2022	62,574	Retrospective Nationwide Observational Study (UK)	Cardiovascular events associated with significantly higher odds of gout flare within the prior 0 to 60 days (204/10 475 [2.0%] vs. 743/52 099 [1.4%]; adjusted OR, 1.93 [95% CI, 1.57–2.38]) and within the prior 61 to 120 days (170/10 475 [1.6%] vs. 628/52 099 [1.2%]; adjusted OR, 1.57 [95% CI, 1.26–1.96])	Gout severity was not controlled for in the analyses. Possible surveillance bias in patients with prior CVD.
	Kobo [65]	2022	344	Single Institution Retrospective Cohort (Israel)	Gout was associated with increased risk for ACS admissions (HR 1.24 95%CI (1.07–1.43), *p* = 0.04)	Data subjected to misclassification and undercoding. Lacks disease severity.
	Huang [66]	2021	3851	Retrospective Nationwide Cohort (Taiwan)	Increased incidence of AMI [adjusted HR 1.36 (95% CI 1.04 to 2.76)]	Based on insurance claims. Lacks potential covariates, including uric acid levels, and unable to obtain confounders from claims dataset.
**Systemic Sclerosis**	Wassif [22]	2022	1738	Nationwide Retrospective Cohort (US–CMS)	SS associated with high risk of mortality, HF, and recurrent MI (adjusted HR 1.49, 95% CI: 1.40–1.58; adjusted HR 1.38, 95% CI: 1.27–1.51; adjusted HR 1.15, 95% CI: 1.05–1.27)	Limited to Medicare patients; may not be generalizable to younger patients. Lacks information on duration, disease-specific activity, laboratory and imaging data, and medications.
	Edigin [67]	2022	1155	Nationwide Retrospective Cohort (US–NIS)	Inpatient mortality for ACS, STEMI, and NSTEMI hospitalizations increased with coexisting SSc 2.02 (95% CI, 1.19–3.43; *p* = 0.009), 2.47 (95% CI, 1.05–5.79; *p* = 0.038) and 2.19 (95% CI, 1.14–4.23; *p* = 0.019), respectively	Data subjected to misclassification and undercoding. Lacks information on cause of inpatient mortality, immunosuppressant or vasodilator compliance, laboratory results, disease duration, disease activity, disease severity, organ involvement, and serological and functional status.
	Cen [68]	2020	14,813	Meta-analysis	Pooled HR for ASCVD was 2.36 (95% CI 1.97–2.81); peripheral vascular disease 5.27 (95% CI 4.27–6.51); myocardial infarction 2.36 (95% CI 1.71–3.25); and stroke 1.52 (95% CI 1.18–1.96) for SS patients	Substantial statistical heterogeneity with regard to the outcomes of stroke and myocardial infarction. Most studies were conducted in Caucasian populations. Possibility of residual confounding.
**Myositis/Dermatomyositis**	Shah [69]	2024	218	Retrospective Cohort Study (US)	Dermatomyositis/polymyositis treated with IMT therapy associated with reduced risk of cardiovascular events (17% vs. 7%; adjusted HR 0.34; 95% CI 0.14–0.82, *p* = 0.02)	Data subjected to misclassification and undercoding. Limited to inpatient outcomes without follow up.
	Wassif [22]	2022	1127	Nationwide Retrospective Cohort (US–CMS)	Dermatomyositis associated with high risk of mortality and recurrent MI (adjusted HR 1.29, 95% CI: 1.19–1.40; adjusted HR 1.19; 95% CI: 1.06–1.33)	Limited to Medicare patients; may not be generalizable to younger patients. Lacks information on duration, disease-specific activity, laboratory and imaging data, and medications.
**Sjogren Syndrome**	Hsiao [70]	2024	948	Nationwide Retrospective Cohort (US–NIS)	Significantly lower in-hospital mortality (adjusted OR: 0.52, 95% CI, 0.36–0.73, *p* < 0.001)	Lacks information on the duration, antibodies, severity, or disease activity of Sjogren syndrome, as well as data on medications prescribed.
**Antiphospholipid Syndrome**	Whittier [49]	2022	1080	Nationwide Retrospective Cohort (US–NIS)	Multivariate analysis of 18–40-year-old SLE strongly associated with ACS hospitalizations (odds ratio, 2.18; 95% confidence interval, 1.546–3.087)	Database relies on billing codes and lacks information on severity, duration of risk factors, or APLS disease activity.
	Cervera [71]	2009	1000	Multicenter Retrospective Study (Europe)	ACS was the cause of death in 19% of 1000 APS patients in a European registry with a five-year observation period	Unable to identify any clinical or immunological parameters. Heterogeneity impacts determining main cause of death.
**Multiple**	Omer [72]	2023	148,428	Meta-Analysis	All-cause mortality was significantly higher in patients with inflammatory rheumatological conditions (RR: 1.12, 95% CI: 1.00 to 1.26, *p*-value: 0.04)	A substantial level of heterogeneity was observed across the analyzed outcomes. Possible confounding factors.
	Wassif [22]	2022	60,072	Nationwide Retrospective Cohort (US–CMS)	All-cause mortality was significantly higher in patients with inflammatory rheumatological conditions (RR: 1.12, 95% CI: 1.00 to 1.26, *p*-value: 0.04)	Limited to Medicare patients; may not be generalizable to younger patients. Lacks information on duration, disease-specific activity, laboratory and imaging data, and medications.
	Weber [73]	2020	2097	Single Center Retrospective Cohort (US)	All-cause mortality and cardiovascular risk higher in systemic inflammatory diseases (adjusted HR 2.41, 95% CI: 1.04–5.61, *p* = 0.041)	Small sample size; unable to control for all baseline characteristics.
	Doornum [74]	2015	1409	Nationwide Retrospective Cohort (Australia)	Increased risk of cardiovascular mortality in autoimmune rheumatic disease at 30 days (OR 1.44 (95% confidence interval (CI): 1.25 to 1.66)) and at 12 months (OR 1.71 (95% CI: 1.51 to 1.94))	Utilized administrative datasets, which have been shown to have inaccuracies. Data subjected to misclassification and undercoding.

**Table 4:** Summary of ongoing randomized trials targeting treatment of inflammation.

Study	Population	Estimated Completion	*n*	Intervention	Primary Outcome
ARTEMIS (NCT06118281)	Acute MI	09/2026	10,000	Ziltivekimab	Composite of cardiovascular death, nonfatal myocardial infarction, nonfatal stroke.
Doxycycline in STEMI (NCT03508232)	STEMI	12/2024	170	Doxycycline 100 mg BID for 7 days	Left ventricular end-systolic volume index
IVORY (NCT04241601)	ACS	01/2023	60	Aldesleukin	Vascular inflammation as measured by FDG PET/CT.
RITA-MI2 (NCT05211401)	Acute MI	04/2027	558	Rituximab	Left ventricular ejection fraction by CMR at 6 months.
